# Decoding the intricacies: a comprehensive analysis of microRNAs in the pathogenesis, diagnosis, prognosis and therapeutic strategies for COVID-19

**DOI:** 10.3389/fmed.2024.1430974

**Published:** 2024-10-07

**Authors:** Shukur Wasman Smail, Sarah Mousa Hirmiz, Akhter Ahmed Ahmed, Niaz Albarzinji, Harem Khdir Awla, Kawa Amin, Christer Janson

**Affiliations:** ^1^College of Pharmacy, Cihan University-Erbil, Kurdistan Region, Erbil, Iraq; ^2^Department of Biology, College of Science, Salahaddin University-Erbil, Erbil, Iraq; ^3^Department of Medicine, Hawler Medical University, Erbil, Iraq; ^4^College of Medicine, University of Sulaimani, Sulaymaniyah, Iraq; ^5^Department of Medical Sciences: Respiratory, Allergy and Sleep Research, Uppsala University, Uppsala, Sweden

**Keywords:** microRNAs, COVID-19, biomarkers, SARS-CoV-2, therapeutic strategies

## Abstract

The pandemic of coronavirus disease-19 (COVID-19), provoked by the appearance of a novel coronavirus named severe acute respiratory syndrome coronavirus-2 (SARS-CoV-2), required a worldwide healthcare emergency. This has elicited an immediate need for accelerated research into its mechanisms of disease, criteria for diagnosis, methods for forecasting outcomes, and treatment approaches. microRNAs (miRNAs), are diminutive RNA molecules, that are non-coding and participate in gene expression regulation post-transcriptionally, having an important participation in regulating immune processes. miRNAs have granted substantial interest in their impact on viral replication, cell proliferation, and modulation of how the host’s immune system responds. This narrative review delves into host miRNAs’ multifaceted roles within the COVID-19 context, highlighting their involvement in disease progression, diagnostics, and prognostics aspects, given their stability in biological fluids and varied expression profiles when responding to an infection. Additionally, we discuss complicated interactions between SARS-CoV-2 and host cellular machinery facilitated by host miRNAs revealing how dysregulation of host miRNA expression profiles advances viral replication, immune evasion, and inflammatory responses. Furthermore, it investigates the potential of host miRNAs as therapeutic agents, whether synthetic or naturally occurring, which could be harnessed to either mitigate harmful inflammation or enhance antiviral responses. However, searching more deeply is needed to clarify how host’s miRNAs are involved in pathogenesis of COVID-19, its diagnosis processes, prognostic assessments, and treatment approaches for patients.

## Graphical abstract

Created with BioRender.com.

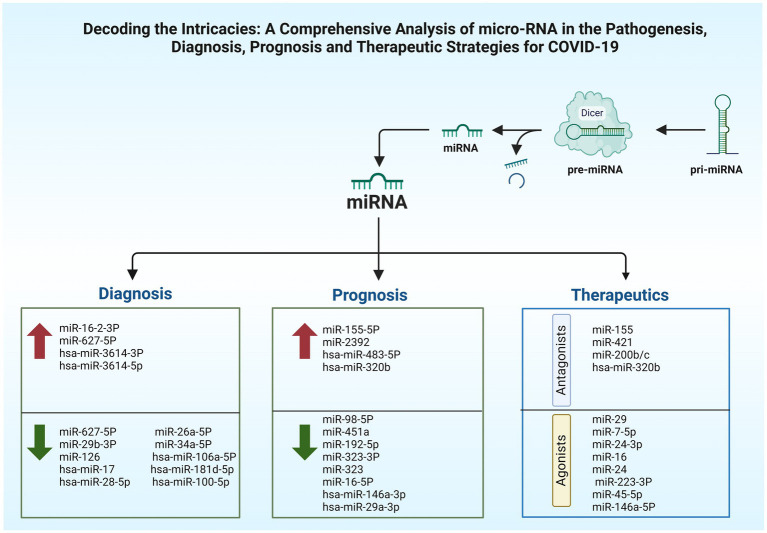

## 1 Introduction

Since its initial appearance in the final quarter of 2019, coronavirus disease-19 which is widely known as COVID-19, having the severe acute respiratory syndrome coronavirus-2 (SARS-CoV-2) as the causative virus, escalating into a health-related global emergency (1). Upon infection, accumulation of viral particles leads to the emergence of variable symptoms, including endothelial lining damage and impaired oxygen diffusion capacity. In the later severe stages, an excessive release of cytokines can contribute to organ failure and potentially result in increased mortality (2). MicroRNAs (miRNAs), which are single-stranded RNAs of 20 to 24 nucleotides, play a role in gene expression regulation by selectively binding to the 3′ untranslated region (UTRs) of mRNAs, resulting in mRNA degradation or translation inhibition, effectively stopping protein synthesis (3). Alterations in the levels of host miRNAs have been noted in individuals who have contracted the SARS-CoV-2 virus (4). Moreover, their close association with various diseases, such as cancer, cardiovascular disorders, and viral diseases, highlights their significance as biomarkers to assess disease severity and monitor treatment responses (5, 6). Human miRNAs play a significant role in regulating cellular activities and their potential interaction with viruses, specifically in the context of SARS-CoV-2 (7). Research has identified certain miRNAs that show significance in binding to essential genes of SARS-CoV-2, making them potential biomarkers for COVID-19 diagnosis (8). Specifically, miR-155-5p, miR-29a-3p, and miR-146a-3p have shown high specificity and sensitivity, indicating their potential as biomarkers for COVID-19 (9).

Genetic and epigenetic factors contribute to the determining the disease severity. Response coming from miRNAs encoded by the host is substantial for prognosticating the SARS-CoV-2 infection’s outcomes (10). In both the acute and post-acute stages of the disease, there were changes in the expression of cellular host miRNAs that make it a good candidate as a marker for disease prognosis (9). The study indicated a notable miR-155-5p elevation in COVID-19 patients that were critically ill, as evidenced by its significant upregulation among 11 host miRNAs assessed for prognostic implications. Additionally, miR-146a-3p and miR-29a-3p show a potential to serve as innovative prognostic indicators for differentiating the acute stages from the post-acute during COVID-19 infection (11).

The clinical significance of host miRNAs is of utmost importance due to their potential therapeutic impact through targeted inhibition, particularly in terms of antiviral effects (12). Host miRNAs have the capacity to augment the body’s protective responses against infections or, at the very least, it could induce pathogen latency (13). The replication/synthesis of viral proteins takes place within the host cell, whereas miRNAs function in preventing the conversion of targeted mRNA into a protein through the translation process. Consequently, miRNAs present themselves as potentially therapeutic approaches to viral diseases (14, 15). This approach can serve as an alternative strategy for managing COVID-19 disease and other related coronavirus illnesses, including utilization of fully complementary miRNAs (cc miRNA) specifically targeting viral genes, suppressing their expression post-transcriptionally. The modified cc miRNAs with a length of 25 to 27 nucleotides, including hsa-miR-2510-3p, miR-3154, hsa-miR-448-3p, miR-5197-3p, miR-7114-5p, hsa-miR-2750-3p, and hsa-miR-1851-5p, demonstrated notable attraction to SARS-CoV-2 genome, suggesting the possibility of an interaction (16). Furthermore, certain viruses possess their own miRNAs, which have the potential to manipulate the signaling pathways of the host, ultimately promoting the virus’s survival and replication. Therefore, the development of drugs that target viral miRNA through inhibition could serve as an additional approach for COVID-19 therapy. Furthermore, research has demonstrated that plant miRNAs that possess identical sequences to human miRNAs could potentially be utilized for COVID-19 treatment (16, 17). The review aims to provide a comprehensive overview of host miRNAs and their involvement in the pathogenesis of SARS-CoV-2 infection. It explores their potential as diagnostic markers to identify different phases of the disease, such as asymptomatic, mild, moderate, and severe cases. Additionally, the review investigates the prognostic value of host miRNAs in predicting unfavorable outcomes, including disease progression, development of complications, and mortality. Furthermore, it delves into the therapeutic relevance of these miRNAs in COVID-19 management, discussing their potential as targets for therapeutic interventions and the development of miRNA-based therapies.

## 2 The emergence and impact of coronaviruses: from SARS-CoV-1 to SARS-CoV-2

Coronaviruses are a heterogeneous group of pathogens responsible for causing illnesses in a wide variety of animal species. Moreover, they are able to cause a spectrum of human respiratory complications, from light to severe (18). Coronaviruses remained relatively obscure in terms of pathologic impact until the early 21st century, when the SARS-CoV-1 emerged in 2002. A subsequent outbreak occurred approximately a decade later with the discovery of the Middle-East respiratory syndrome coronavirus (MERS-CoV) in 2012. Both viruses are believed to have a zoonotic origin, with SARS-CoV-1 being linked to bats and palm civets and MERS-CoV to dromedary camels. These coronaviruses are well known for their ability to cause severe and frequently lethal respiratory diseases (19). An outbreak of pneumonia caused by an unknown agent was announced in Wuhan city of China in the final quarter of 2019. Subsequent investigations involving the isolation of the virus from patients with acute pneumonia, accompanied by RNA metagenomic sequencing, uncovered a novel beta coronavirus as the responsible pathogen (18). The SARS-CoV-2 emerged as a new coronavirus, and within a period of 6 months, it was reported that 215 nations were affected with a mortality rate of approximately 750 thousand humans (20). SARS-CoV-2 seemed to have a quite similar genetic sequence and structural resemblance to SARS-CoV-1 of 2002 (21). SARS-CoV-2 is highly infectious and transmissible SARS-CoV-2 virus that belongs to *Orthocoronavirinae* subfamily, the virus led to a global spread of COVID-19, an illness causing severe respiratory complications, which put the health and safety of the public at risk (22).

### 2.1 The SARS-CoV-2’s biology and its replication cycle

**Figure 1:**
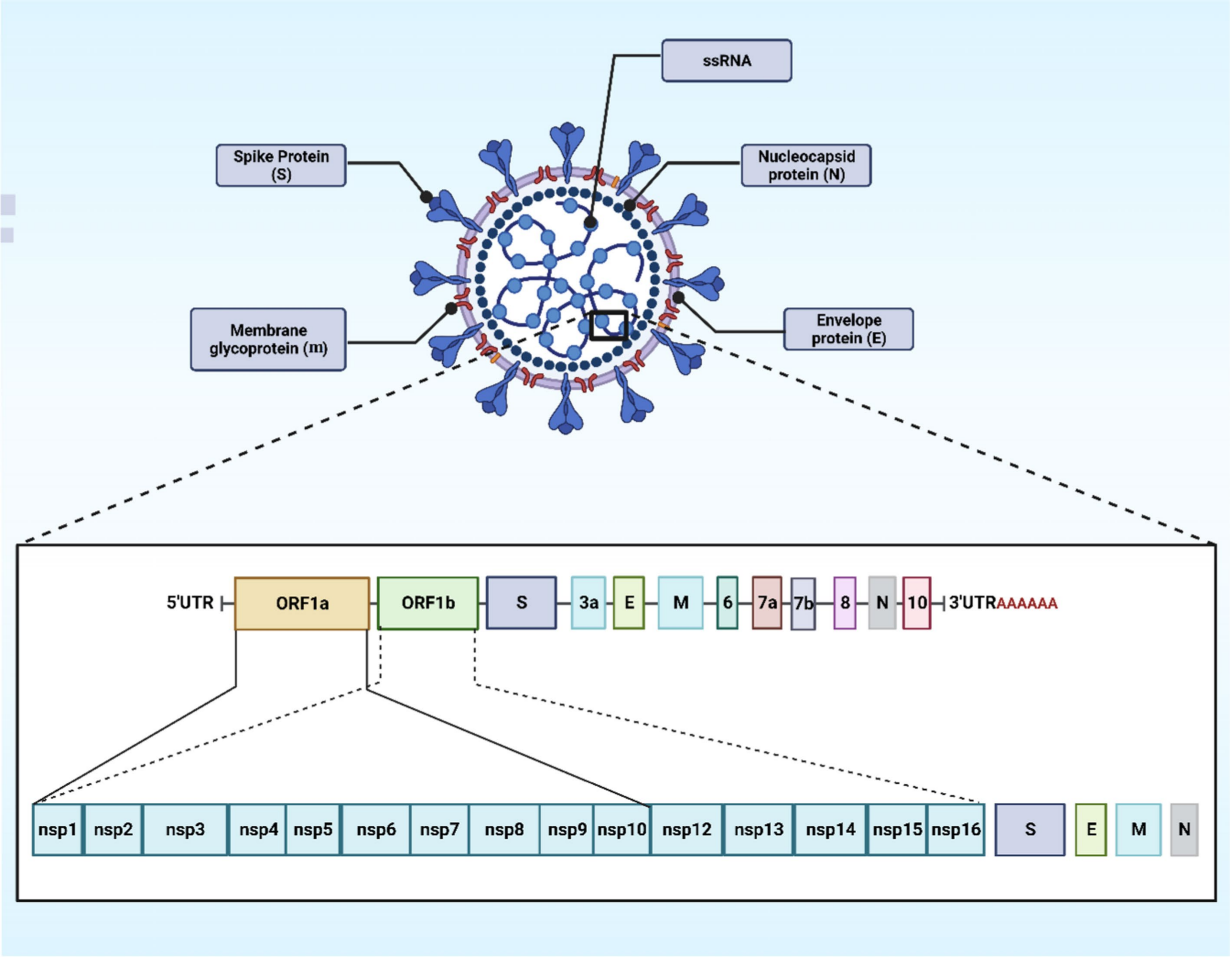
The schematic structure and organization of the SARS-CoV-2 genome. The image was created using the BioRender program, available at https://www.biorender.com.

Coronaviruses contain the largest RNA genome compared to all the other RNA viruses, reaching a length of 27–32 kb. The positive-sense single-stranded RNA (+ssRNA) acts like a messenger RNA that can be translated directly upon host cells’ invasion (23). In SARS-CoV-2 viruses, the nucleocapsid (N) protein encases the viral RNA to form the N structure and is integral to virus replication and mRNA transcription. This N protein also disrupts cell structures and dampens immune responses by shielding viral RNA from the host’s RNA interference pathway, which typically destructs such RNA. It is triggering of antibody and cell-mediated immune reactions post-infection highlights its significance in both diagnostic and vaccine research (24). An envelope with the three associated structural proteins on its surface further surrounds the capsid. The envelope is a lipid bilayer that can be broken down using soap. This viral envelope is derived from the membrane of the host cells (23). Looking at coronaviruses, it is obvious that they have a series of glycoprotein spikes on their envelope. The spiky projections give the appearance of a crown under an electron microscope examination, hence, the given name (corona) from the Latin word *coronam* for crown (25). Four essential structural proteins are present in SARS-CoV-2 virus, spike (S) proteins, membrane (M) proteins, envelope (E) proteins, and nucleocapsid (N) proteins. Non-structural proteins (nsps) are present as well, which are 16 in number (nsp 1–16) (23). The S protein is a glycoprotein that the SARS-CoV-2 virus utilizes as a unique surface antigen. The significance of this protein in attaching to and invading host cells, and in disease progression, renders it a pivotal focus for devising vaccines and therapeutics against COVID-19 (26). SARS-CoV-2’s S proteins encourage the binding and following penetration of the virus into host cells subsequent to the identification of angiotensin-converting enzyme 2 (ACE2) receptors, which are ubiquitously found in many organs like the heart, lungs, and kidneys. The S glycoprotein is bifunctional, having two subunits (S1 and S2). The S1 facilitates the attachment to the ACE2 receptor, while the S2 mediates viral fusion with the host’s cell membrane, thus provoking infection (26). The M glycoprotein represents the most abundant structural protein discovered in coronaviruses. The rest of the structural proteins are bound to the M proteins. This adhering aids in the finalization of viral assemblage process through stabilizing and complex forming between RNA and N protein (27). The E protein, despite its modest dimensions, is pivotal in maintaining the structural integrity of viral particles by its interaction with the M protein (Figure 1).

**Figure 2:**
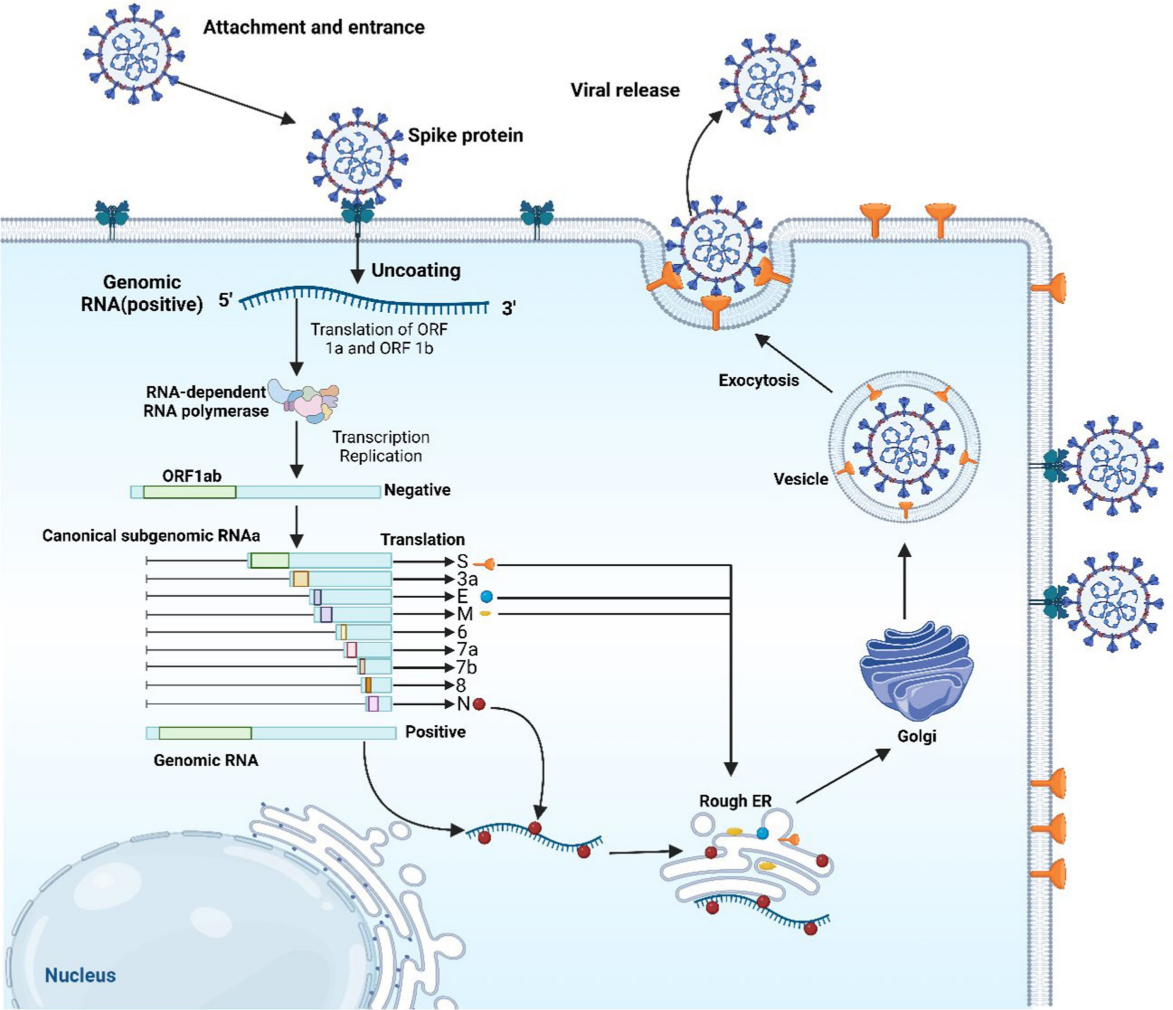
The replication cycle of SARS-CoV-2. The image was created using the BioRender program, available at https://www.biorender.com.

E protein was found to have high expression in cells infected with the virus, having an important role in the assemblage and egress of the new viruses. Removing E protein could notably reduce or completely stop the virus’s disease induction potentiality, due to its involvement in the life cycle of the virus (28, 29). SARS-CoV-2 begins infecting cells by its S proteins latching onto ACE2 receptors, followed by the action of priming by transmembrane serine protease 2 (TMPRSS2) and cathepsin L (Figure 2). Within the cellular environment, the virus releases its RNA, which is utilized for the synthesis of replication proteins such as RNA-dependent RNA polymerase in vesicles derived from the endoplasmic reticulum (ER). The replicated RNA generates more viral components and genomes. New viruses exit cells and the infection start spreading after the assemblage of the viral structural proteins (N, S, M, and E) within the ER-Golgi intermediate compartment (ERGIC) (30).

### 2.2 Immunological response to SARS-CoV-2 infection

Antigen-presenting cells (APCs) like macrophages and dendritic cells (DCs) having an integral role in the activation of innate and adaptive immune responses, are able to detect the virus’s viral antigen as it enters into host cells, by their diversified pattern recognition receptors (PRRs), that encompasses NOD-like receptors, RIG-I-Like receptors (RLRs), and Toll-like receptors (TLRs), the latter is the most significant type of PRR, mostly occurring on macrophage cells. TLRs is a group of transmembrane receptor proteins which recognize pathogen-associated molecular patterns (PAMPs). TLRs group with its 10 members occupy different places, TLR (1, 2, 4, 5, 6, and 10) are found on cell surface, while TLR (3, 7, 8, and 9) are found located in intracellular compartments such as endosomes (31, 32).

**Figure 3:**
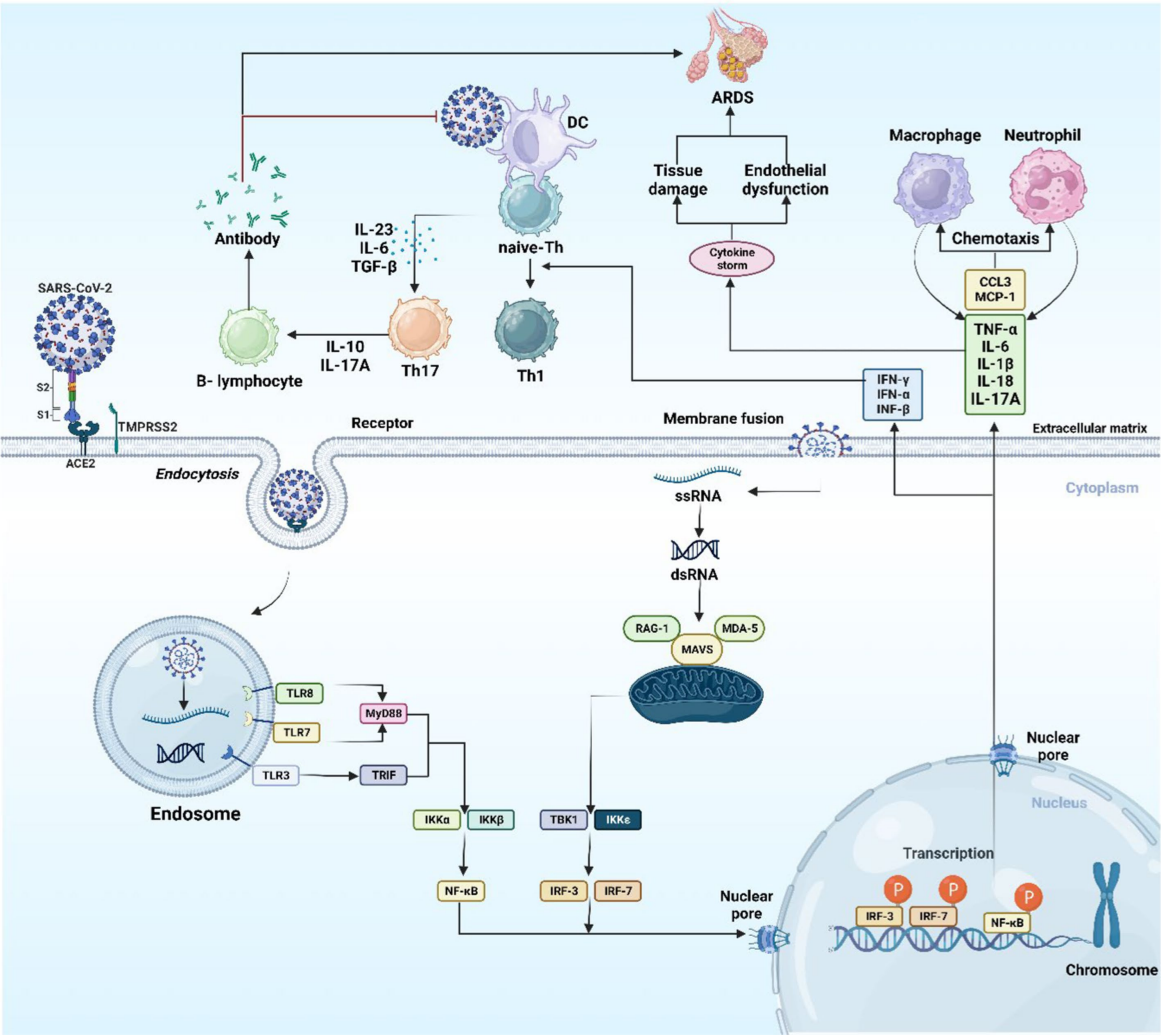
The host immune response to SARS-CoV-2. The image was created using the BioRender program, available at https://www.biorender.com.

Once the virus is identified by TLR4 of the APC, the viral antigenic peptide is presented through class II of major histocompatibility complex (MHC) to T-helper (Th) cell, while class I of MHC represent the antigenic peptide to cytotoxic T-lymphocyte (CTL). APC releases interleukin (IL)-12, a co-stimulatory molecule, which additionally assists in the activation of Th1. For the successful eradication of a virus, the stimulation of Th1 by IL-12 and MHC class I antigen presentation is required (33, 34). When SARS-CoV-2 interacts with PRRs, interferon regulatory factor (IRF) and nuclear factor-kappa B (NF-κB) are generated and transported to the nucleus. The transcription of inflammatory cytokines [IL-1, IL-6, and tumor necrosis factor-α (TNF-α)] and interferons (IFNs) like (IFN-α, IFN-β, and IFN-γ) is facilitated by NF-κB and IRF, respectively. The anti-viral response is attributed to IFNs, which increase the cell’s immunity to viral infection (Figure 3) (35).

The immune system relies on neutrophils to ward off infections. During the primary phases of COVID-19 infection, neutrophils dispatch to the infected area helping in controlling viral replication and impeding the virus’s spread. During advanced severe cases of COVID-19, it is evident that the body’s immune defenses might become disrupted, leading to the cytokine storm (CS). As a result of this, high level of cytokines and other inflammatory molecules are released, which can cause harm to lungs and other organs. It is proposed that neutrophils contribute to the emergence of CS in COVID-19, as they can release pro-inflammatory molecules, causing tissue damage. Additionally, it is indicated that persons with COVID-19 in their severe stages show elevated levels of circulating neutrophils in comparison with less severe cases (36).

## 3 The significance of microRNAs and their biogenesis

**Figure 4:**
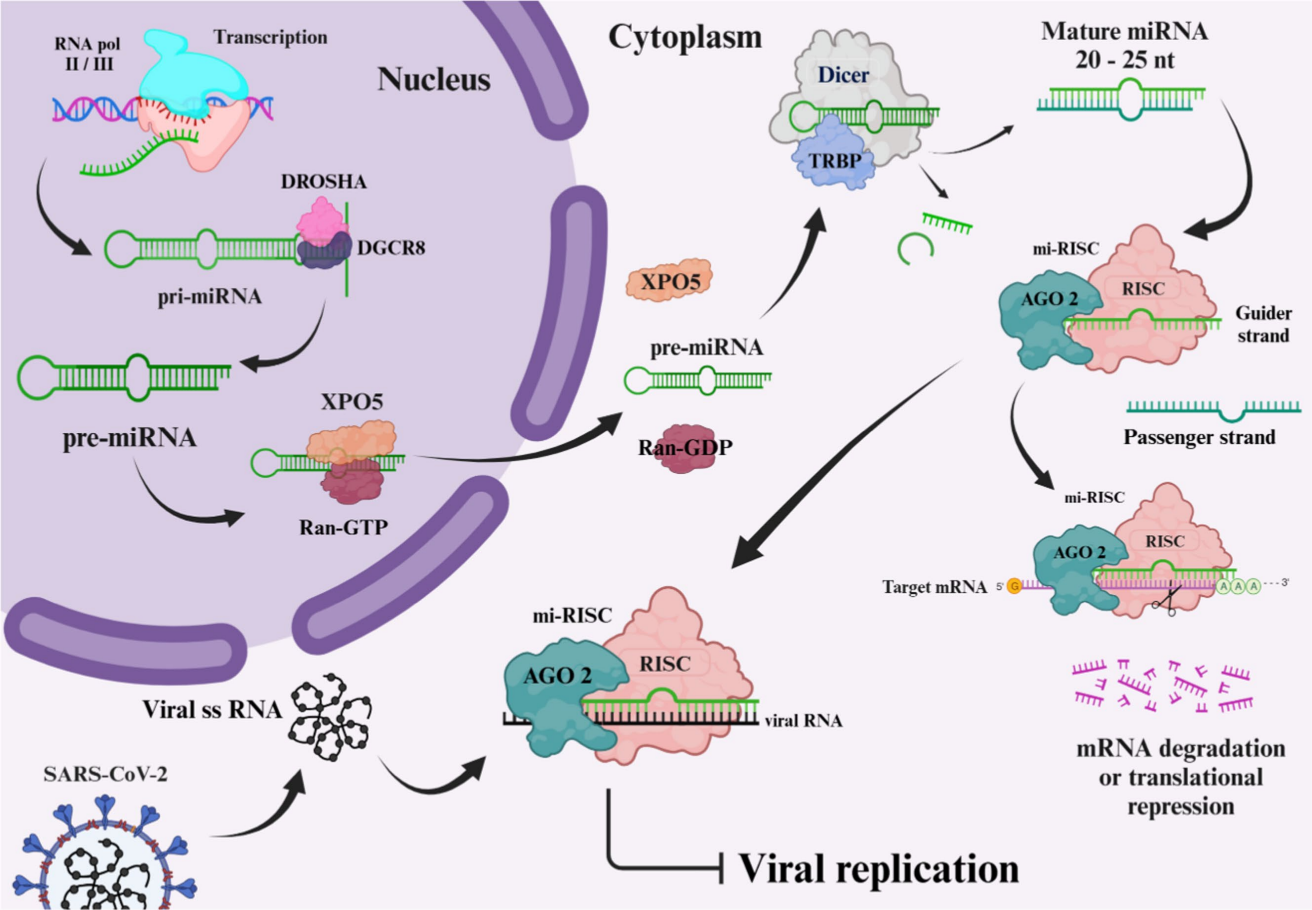
Biogenesis of microRNA. The image was created using the BioRender program, available at https://www.biorender.com.

microRNAs (miRNA), typically ranging from 20 to 24 nucleotides, represent a subset of non-coding RNAs that are instrumental in controlling gene expression post-transcriptionally. These small non-coding RNAs are critical for the modulation of gene activity (37). Current research has documented approximately 2,300 mature miRNAs in human beings, and an estimated half of these miRNAs are listed in the database named miRbase (38). While the majority of these miRNAs remain within cells, a category, referred to as circulating miRNAs, are measurable extracellularly in variable bodily fluids including plasma, cerebrospinal fluid (CSF), tears, and saliva. Circulating miRNAs, carried via extracellular vesicles in association with RNA-binding protein complexes, are featured by their outstanding resilience in extreme conditions, in addition to their robustness against degradation by native RNase. Therefore, they are proposed as promising biomarkers (39). miRNAs’ biogenesis is a sophisticated, multi-sites and steps pathway, beginning within the nucleus, and finalizing structures out in the cytoplasm. The pathway relies on diverse enzymes and protein structures sequentially operating together to yield an operational mature miRNA. Among several synthesis routes, Figure 4 illustrates the canonical pathway, which is being thoroughly investigated. The pathway begins with the transcription of DNA sequences. It is worth mentioning that approximately half of the miRNAs associated with humans, are encoded by introns of genes encoding proteins (40, 41). RNA polymerase (RNA pol) II or III carries out the transcription of DNA regions, supplying the primary miRNA (pri-miRNAs), shaped as long, hairpin, double stranded RNAs, equipped with a 5′ cap and a 3′ poly-A tail having a length of 70 nucleotides, pri-miRNAs have the capacity to provide up to six miRNA precursors. Initial processing of pri-miRNAs initiates in the nucleus, via an enzymatic complex encompassing of RNase III drosha and DiGeorge syndrome critical region (DGCR8) as the assisting protein. The latter complex adheres to pri-miRNA molecule, cleaving the cap and polyadenylated tail, shaping it as shorter precursor miRNA (pre-miRNA). Exporting the pre-miRNA is done in association with exportin-5 (XPO5) (39, 41, 42). Exported to the cytoplasmic area, dicer enzyme finalizes shaping the pre-miRNAs into their mature form by removing the unpaired bases from the pre-miRNA molecule, leaving it as a duplex with a 21 to 25 nucleotides in length. Phosphorylated 5′ end 3′ ends that each overhang by two nucleotides are featuring this mature structure. Mature miRNAs serve as integral components of the RNA-induced silencing complex (RISC), comprising the incorporation of argonaute protein, including AGO2. During the process of mi-RISC assembly, the double-stranded miRNA gets separated, passenger strand gets discarded, retaining the guider strand to guide the complex. Guider strand navigates the mi-RISC complex to the targeted mRNA, base pairing results in the expression repression of the gene, either by mRNA degradation, or restraining the translation stage (39, 41, 43, 44). The binding flexibility of miRNA molecules with various mRNA sequences is due to the small number of nucleotide bases involved in the miRNA-mRNA interaction. A lower extent of base pairing can diminish protein production capacity from the mRNA, whereas a more substantial base pairing can instigate the breakdown of the mRNA (39). Recent research reveals that host miRNAs can influence the infectious cycle of RNA viruses by binding to their genomes, thus affecting viral replication and pathogenesis. Additionally, viral infection may prompt shifts in host miRNA levels, altering gene expression in ways that could favor the virus or enhance the host’s antiviral response, potentially restraining viral spread (45).

### 3.1 Role of microRNAs in COVID-19

**Figure 5:**
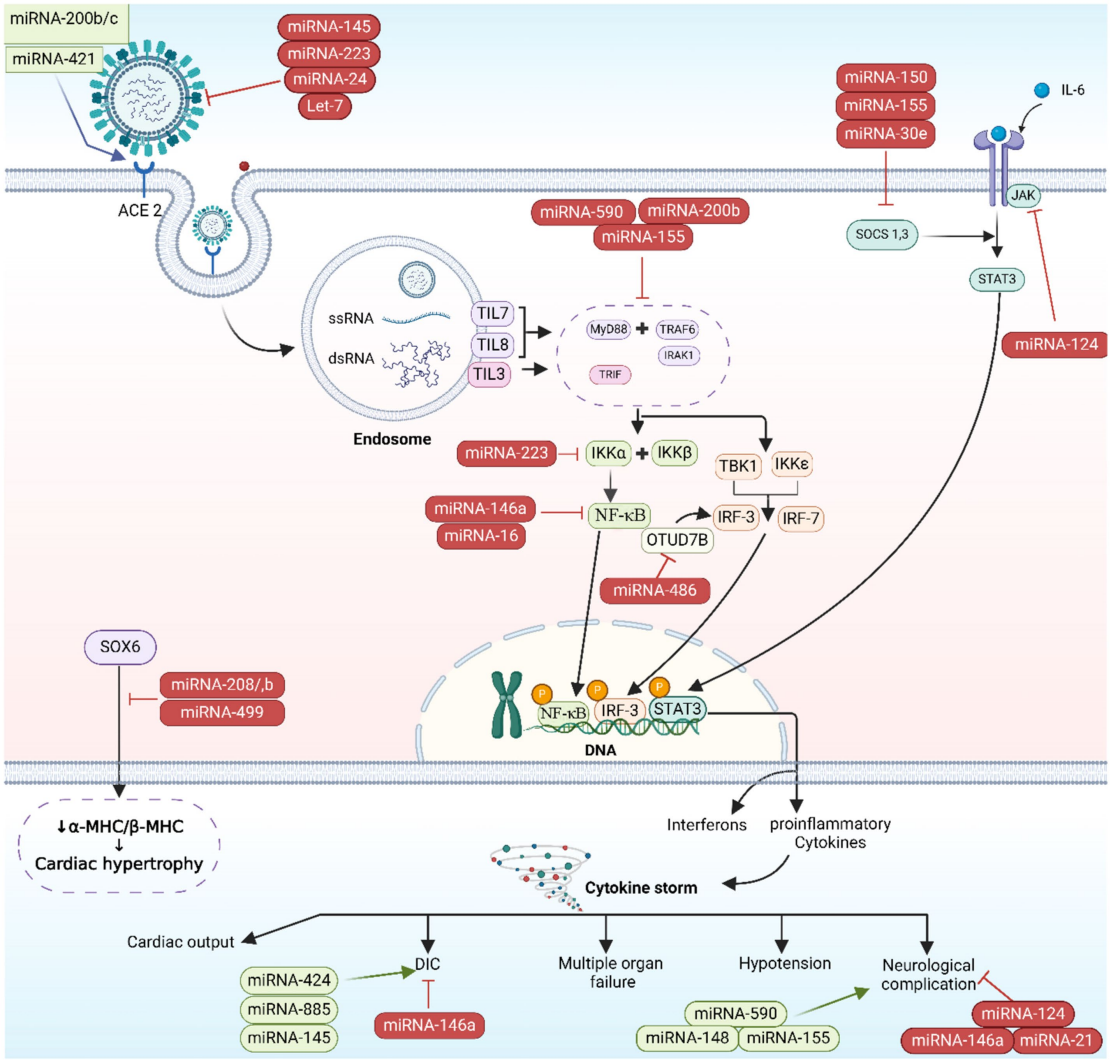
microRNA-induced immunopathogenesis. The image was generated utilizing the BioRender software, accessible at the website https://www.biorender.com.

Cellular injury and the potential disruption of vital organs’ functionality, these include liver, lungs, brain, gut tissues, and kidneys, can be induced as the SARS-CoV-2 virus elicits an immunological and inflammatory responses (3). CS, a deep inflammatory response that follows up the primary infective stage, is initiated by the activation of inflammation-related genes including IL-6, IL-8, granulocyte colony-stimulating factor (G-CSF), and NF-κB proteins. CS initiates as the S proteins of SARS-CoV-2 bind to ACE2 receptors, provoking ACE/angiotensin II (Ang II)/angiotensin II type I receptor (AT1R) pathway giving rise to a heightened activation of NF-κB by IL-6/signal transducer along with activator of transcription (STATs) pathway (Figure 5) (46). CS can lead to multiple organ failures and may cause fatality (47). Host miRNAs are pivotal in the immune response during viral infections, as they fine-tune cytokines production. By enhancing or suppressing cytokine signaling, miRNAs can either boost beneficial immune functions or restrain detrimental inflammatory responses, thereby preserving the immune system’s equilibrium (48).

#### 3.1.1 The involvement of microRNAs in the immune response to COVID-19

In post-transcriptional gene expression, miRNAs play a vital role and affect several biological processes, such as inflammation and immune responses. Two types of miRNAs exist: pro-inflammatory and anti-inflammatory miRNAs. These miRNAs, miR-155, and miR-92a, have pro-inflammatory effects. TLR activation leads to the secretion of multiple transcription factors such as activator protein (AP-1), STATs, and (purine-rich box) PU.1, which lead to upregulate miR-155. Inhibition of SH2 containing inositol 5′ phosphatases 1 (SHIP1) and suppressor of cytokine signaling 1 (SOCS1) by miR-155 results in the release of IL-6 and IFNs, respectively (49). The suppression of SOCS1 by miR-155 leads to the activation of Janus kinase 2 (JAK2)/STAT1 and TLR/NF-κB signaling pathways of IFN-γ, resulting in macrophage 1 polarization (50). Upregulation of hypoxia-inducible factor-1 alpha (HIF-1α) and TNF-α, as well as downregulation of heme oxygenase-1 (HO-1), contribute to miR-155’s enhancement of lipopolysaccharide (LPS)-induced lung injury (51). Additionally, miR-92a triggers inflammation and lung damage by activating the phosphatase tensin homolog (PTEN)/serine/threonine protein kinase (Akt)/NF-κB signaling pathway. Therefore, inhibiting the PTEN/Akt/NF-κB signaling pathway through miR-155 antagonism helps alleviate lung injury (52). On the other hand, miR-146a and miR-124 exhibit anti-inflammatory properties. miR-146a blocks TLR, IL-1 receptor associated protein kinase 1 (IRAK1), and TNF receptor-associated factor 6 (TRAF6) to reduce inflammation, while miR-124 inhibits IL-6 and TNF-α release by targeting TNF-α converting enzyme (TACE) and STAT3 pathway (53).

The severity of COVID-19 is increased by the activation of NLPR3 inflammasome and the inhibition of vascular endothelial growth factor (VEGF), both of which are linked to miR-205-5p (54). The expression of NLPR3 inflammasome and inflammatory cytokine (IL-1β) can be decreased by miR-205-5p through the inhibition of B-cell lymphoma 6 (BCL6) (55). The study found that miR-205-5p blocks VEGF, a growth factor induced by the hypoxia-causing virus. Hypoxia leads to an increase in HIF-1 levels, causing higher VEGF levels and pulmonary edema. It is believed that miR-205-5p reduces lung edema by suppressing the HIF-1/VEGF pathway (56).

The expression of the miR-143-3p gene influences the number of neutrophils by affecting the expression of the *BCL2* gene (57). miR-143-3p boosts neutrophil count through multiple mechanisms, including increasing the expression of cell adhesion molecules that facilitate neutrophil transmigration (58). The second mechanism involves miR-143-3p inhibiting BLC2, an anti-apoptotic protein. This particular miRNA hinders neutrophil apoptosis through this specific mechanism. By enhancing myeliod cell leukemia-1 (Mcl-1) levels and activating the phosphoinositide 3 kinase (PI3K)/Akt/STAT pathway, miR-143-3p promotes neutrophil survival and prolongs their lifespan (59).

The miR-19b-3p molecule attaches to specific regions of the SARS-CoV-2 virus, boosting the activity of CTLs (38). The reason behind increase activity of CTL by miR-19b-3p is due to upregulate of PTEN. In addition, PTEN also plays a significant part in CD4^+^ and CTL cell development. PTEN deficiency can lead to defects in the development of these T cell subsets, affecting their maturation and functionality. PTEN within the T cell lineage has diverse roles, including negative regulation of TCR and CD28 signaling, involvement in T cell development, influence on Treg development, and participation in immune checkpoint inhibitory signaling pathways. Understanding the multifaceted functions of PTEN in T cells is vital for unraveling the complexities of T cell biology and may have implications for therapeutic interventions in various immune-related disorders (60).

Decreasing the level of miR-155 in the serum severely impacts the ability of CTL (61) to respond to the virus, resulting in a reduced level of CTL and their ability to eliminate the virus (62). miR-155 plays a crucial role in the generation and function of CTL during viral infections. Further studies have shown that miR-155 regulates key pathways involved in CTL activation and effector functions. For example, it has been demonstrated that miR-155 targets SHIP-1, a negative regulator of T cell receptor signaling, leading to enhanced T cell activation and proliferation. Additionally, miR-155 has been shown to target SOCS1, a suppressor of cytokine signaling, resulting in increased production of pro-inflammatory cytokines by CTL (63).

#### 3.1.2 Endogenous players confronting SARS-CoV-2 viruses

Human related antiviral cellular responses are enhanced by miRNAs, bringing about the disruption of vital pathways related to viral replication, these include PI3K/Akt and p38 mitogen-activated protein kinases (MAPK). miRNAs like hsa-miR-17-5p, hsa-miR-20b-5p, and hsa-miR-323a-5p have participated in SARS-CoV-2 proliferation inhibition in host cells (64). miR-146a, as an early-response miRNA to viral infections, it has the potentiality to both target genome of SARS-CoV-2 and regulate TLRs signaling in order to moderate inflammation, as it is known that TLRs are important in viral detection and subsequent innate immunity stimulation, enhancing the release of inflammation related cytokines such as IL-1, IL-6, TNF-α, and type I IFNs. The over-stimulation of immune responses is prevented through TLRs regulation (65, 66). miR-146a influencing cardiac regions, miR-4262 located in pulmonary cells, and miR-18 beside miR-125b found in renal tissues, are certain miRNAs identified as fundamental regulators of ACE2 genes expression (67). In a study, bioinformatics analysis results using RNA hybrid suggested that 125 miRNAs out of 240 correlated to aging have targeting ability of SARS-CoV-2’s genome, providing potential antiviral influence. Particularly, it is believed that miR-24-3p, miR-7-5p, miR-145-5p, and miR-223-3p have interaction with S proteins of SARS-CoV-2, as it is known that S proteins facilitate the invasion of host cells by the virus, also, these four miRNAs were low in level in old individuals, besides miR-223-3p, miR-24-3p, and miR-7-5p mostly lowered in diabetic persons. Furthermore, both circulatory exosomal miRNAs and synthetic ones were indicated to suppress the S proteins, thus potentially hindering SARS-CoV-2 replication (68). Table 1 further depicts native miRNAs’ influence during COVID-19 infection.

**Table 1:** microRNAs involved in COVID-19 pathogenesis.

Circulatory miRNAs	Expression	Targets	Human/animal/cells	Clinical sample	Studying groups/cell lines	Role	Source
miR-146a	↓	TRAF6	Human	Serum	Critical illness (*n* = 21) Severe illness (*n* = 20) Moderate illness (*n* = 20) Mild illness (*n* = 21) Asymptomatic illness (*n* = 21) Control group (*n* = 20)	Anti-neuroinflammation action	(148)
hsa-miR-17-5p	↓	—	Human	Peripheral blood	COVID-19 patients: (*n* = 10) Control group (*n* = 4)	Regulates immune responses and viral replication during infection	(109)
miR-146a	↑	TRAF6, IRAK1	Human cell line	Human monocytes	Acute monocytic leukemia (THP-1) cell line	Inflammation moderation and cytokine signaling can be controlled through the regulation of TLRs signaling	(149)
miR-7-5p miR-24-3p miR-145-5p miR-223-3p	↓	S proteins	Human	Serum exosomes	A group of different age (3 young, age <30 and 10 elderly persons, age >60) Diabetic group (*n* = 15)	Inhibit S protein expression to prevent SARS-CoV-2 multiplication	(68)
miR-146a	↑	IRAK1, TRAF6, and proinflammatory cytokines (IL-6, and IL-8)	Human	Serum	Moderate COVID-19 (*n* = 22) Severe COVID-19 (*n* = 15) Control group (*n* = 15)	Regulation of inflammatory response	(75)
miR-146b	↑	IRAK1, TRAF6, and proinflammatory cytokines (IL-6, and IL-8)	Human	Serum		Regulation of inflammatory response	
miR-21	↑	IL-12	Human	Serum		Regulation of inflammatory response	
miR-499	↑	SOX6	Human	Serum		Regulation of inflammatory response	
miR-155	↓	SHIP1 and SOCS	Human	Serum		Regulating inflammation and antiviral reactions	
miR-486-5p	↓	OTUD7B	Human	Plasma	Study (1) Critical COVID-19 (*n* = 36) Moderate COVID-19 (*n* = 79) Study (2) ICU COVID-19 survivors (*n* = 20) ICU COVID-19 non-survivors (*n* = 16)	By modulating antiviral responses, it prevents acute lung damage	(119)
miR-34a	↑	—	Human	Peripheral blood mononuclear cells	Study (1) Control group (*n* = 50) Moderate COVID-19 (*n* = 50) ICU COVID-19 patients (*n* = 50) Study (2) Diabetic COVID-19 group (*n* = 30) Non-diabetic COVID-19 group (*n* = 70)	Potential indicator for differentiating diabetic COVID-19 patients	(100)
miR-21 miR-155 miR-208a miR-499	↑	—	Human	Serum	Discovery study COVID-19 patients (*n* = 18) Control group (*n* = 15) Validation study COVID-19 patients (*n* = 20) Influenza-ARDS group (*n* = 13) Control group (*n* = 32)	Predictive factors for inflammation-induced myocardial damage	(90)
miR-148a miR-590	↑	USP33, IRF9, NF-κB, TNF-α, and IFN-β	Human cell line	Exosomes released from spike transfected cells	HEK-293 T and human microglial cell line (CHME3)	Initiate inflammation in the central nervous system	(95)
miR-15b-5p	↑	SARS-CoV-2 genome, IFN-γ, and CD69	Human	Whole blood	Moderate COVID-19 (*n* = 6) Severe COVID-19 (*n* = 6) Control COVID-19 (*n* = 4)	Facilitates the replication of RNA viruses and increased the severity of COVID-19; caused T-cell depletion	(123)
miR-155-5p	↑	CLDN1	Human cell line	Human cell TIME	Cell line TIME (ATCC^®^ number CRL-4025) served as an *in vitro* representation of microvascular endothelial cells	Persistent neuropathic pain in long COVID-19 by expression reduction of CLDN1 levels and consequently increasing barrier permeability	(4, 106)
miR-145 miR-885	↓	Tissue factor, and von Willebrand factor	Human	Circulating exosome from serum	COVID-19 patients (*n* = 26) Control group (*n* = 10)	Prevent blood clotting occurrence in COVID-19 cases	(93)
miR-424-5p	↑	—	Human	Serum exosomes	A group with different age (3 young, age <30 and 10 old, age >60)	Links to thrombosis	(150)
miR-200a	↓	SMAD-3, matrix proteins, TGF-dependent ETM, TGF-2	Animal cell line/animal model	Rat proximal-tubular epithelial cells	*In vitro* study NRK52E cell line, which is derived from rat kidney tubular epithelial cells *In vivo* study Kidney fibrosis models (early and late stages)	Protects the kidney from fibrosis	(151)
miR-141	↓	—					
Let-7b	↓	TGFBR1	Animal cell line/animal model	Rat proximal-tubular epithelial cells	*In vitro* study NRK52E cell line, which is derived from rat kidney tubular epithelial cells *In vivo* study Diabetic nephropathy models (early and advanced stages) Non-diabetic adenine induced renal fibrosis model	Prevents Renal fibrosis	(152)
miR-216a	↑	YBX1	Animals	Cell culture-primary mouse mesangial cells	Renal mesangial cells	Diabetic nephropathy	(153)
miR-208a	↑	β-MHC mRNA	Human tissues	Endomyocardial biopsy	DCM patients (*n* = 82) Control group (*n* = 21)	Elevates levels of medl3 expression within the heart contributes to an increase in the expression of β-MHC	(38, 154)
miR-15b-5p	↓	PTEN-PI3K/Akt pathway	Human cell line	Human kidney cells	HK-2 cell line	Inhibits renal cell apoptosis induced by high glucose levels	(99)

Akt, serine/threonine protein kinase; ARDS, acute respiratory distress syndrome; CD, cluster of differentiation; CLDN1, claudin 1; COVID-19, coronavirus disease-2019; DCM, dilated cardiomyopathy; EMT, epithelial-mesenchymal-transitions; hsa, homo sapiens; ICU, intensive care unit; IL, interleukin; IRF, interferon regulatory factor; IFN-β, interferon beta; IRAK1, interleukin-1 receptor-associated kinase 1; miR, microRNA; MHC-β, myosin heavy chain beta; NF-κB, nuclear factor-kappa B; PI3K, phosphatidylinositol 3 kinase; PTEN, phosphatase tensin homolog; OTUD7B, OTU domain-containing protein 7B; S, spike; SARS-CoV-2, severe acute respiratory syndrome-coronavirus-2; SHIP1, SH2 inositol 5-phosphatase 1; SMAD, suppressor of mothers against decapentaplegic; SOCS, suppressor of cytokine signaling; SOX6, SRY-box transcription factor 6; TGF, transforming growth factor; TGFBR, transforming growth factor beta receptor; TNF, tumor necrosis factor; TLRs, Toll-like receptors; TRAF6, TNF receptor-associated factor 6; USP33, ubiquitin specific peptidase 33; YBX1, Y-box binding protein-1.

##### 3.1.2.1 Influence of native microRNAs on COVID-19 severity

Circulatory miRNAs, ubiquitously circulate within nearly all bodily fluids, possessing the ability to serve as highly sensitive biomarkers to precisely reflect the physiological and pathological conditions of the body. Encased within extracellular vesicles, circulatory miRNAs are implicated in facilitating communication between immune cells and may offer valuable insight into disease mechanisms as biomarkers (69, 70). The extracellular vesicles are lipid bilayer-bound particles released by various cell types, including immune cells. These extracellular vesicles are taken up by recipient cells through endocytosis or fusion with the plasma membrane, leading to the delivery of miRNAs into the cytoplasm of the recipient cells. For example, miR-21 and miR-155 are known to be carried by exosomes derived from dendritic cells and can modulate the activity of T cells, influencing their differentiation and function (71, 72). By influencing adhesion molecule levels, miRNAs affect the efficiency of immune cell migration to the inflammatory sites. miRNAs can regulate the expression of adhesion molecules, which allow immune cells to attach to blood vessel walls and migrate into tissues (73). miR-146a has been shown to target TRAF6, which may further down-regulate the IL-17/intercellular adhesion molecule 1 (ICAM-1) pathway. Thus, miR-146a inhibit the expression of ICAM-1, an adhesion molecule, thereby reducing the migration of monocytes to inflammatory sites (74).

Host’s miRNAs play a significant role in managing cytokines activities in the inflammatory phase of COVID-19. In a study, miRNAs in hematologic specimens taken from COVID-19 hospitalized cases were analyzed and compared to non-infected individuals to evaluate their potential as a mortality prediction biomarker. Inflammatory gene regulation was shown to be influenced by specific miRNAs: Cytokine gene expression is managed when miR-146 targets TRAF6 and IRAK1, and modulates the pro-inflammatory cytokines (IL-6 and IL-8) levels. miR-21, miR-499, and miR-155 were shown to regulate IL-12, target SRY box 6 (SOX6) gene to influence sepsis-related lung deterioration, and down-regulate SHIP1 and SOCS1, respectively. SHIP1 and SOCS1 are known to serve as inhibitors of inflammatory reactions in macrophages (Figure 5). The same study concluded that miR-155 can be used to forecast survival and prognosis in patients after contracting SARS-CoV-2 (75). The relationship between miR-21, miR-499, miR-155, and IL-12 targeting SOX6 is complex and not completely understood. It is possible that the influence of these miRNAs on IL-12 production is indirect, occurring via their targeting of SOX6 (76). The transcription factor SOX6 can impact the expression of inflammation-related genes like IL-12 (77–79). In addition, miR-155 indirectly regulate IL-12 production by downregulating SHIP-1 as miR-155 has recently been shown to suppress SHIP-1 expression by binding to its 3′ untranslated region (80, 81). When SHIP-1 is not present, macrophages produce higher levels of IL-12 (82). Moreover, the miRNAs regulate IL-12 by targeting SOCS1 (75). Infections efficiently stimulate SOCS1 expression in macrophages. The reported inhibition of STAT mediated responses by SOCS1 is responsible for the regulation of IL-12 expression in macrophages. The biological function of numerous cytokines is mediated by STAT, and SOCS1 plays a regulatory role in IL-12 (50, 83) and eventually the miRNAs exert their effect on IL-12 by SOCS (38, 84). SHIP-1 has been found to act as a suppressor of hematopoietic transformation by blocking the PI3K/Akt signaling pathway. SHIP1 is a target of miR-155 in which miR-155 decrease the expression of SHIP-1 and it is known that SHIP-1 is a suppressor of hematopoietic transformation as its activity can inhibit the PI3K/Akt signaling pathway in acute myeloid leukemia and myelodysplastic syndromes. Therefore, these findings indicate that miR-155/SHIP-1/Akt pathways may be valuable clinical biomarkers for disease progression, even in COVID-19 (85, 86).

###### 3.1.2.1.1 The involvement of microRNA in pulmonary injury

**Figure 6:**
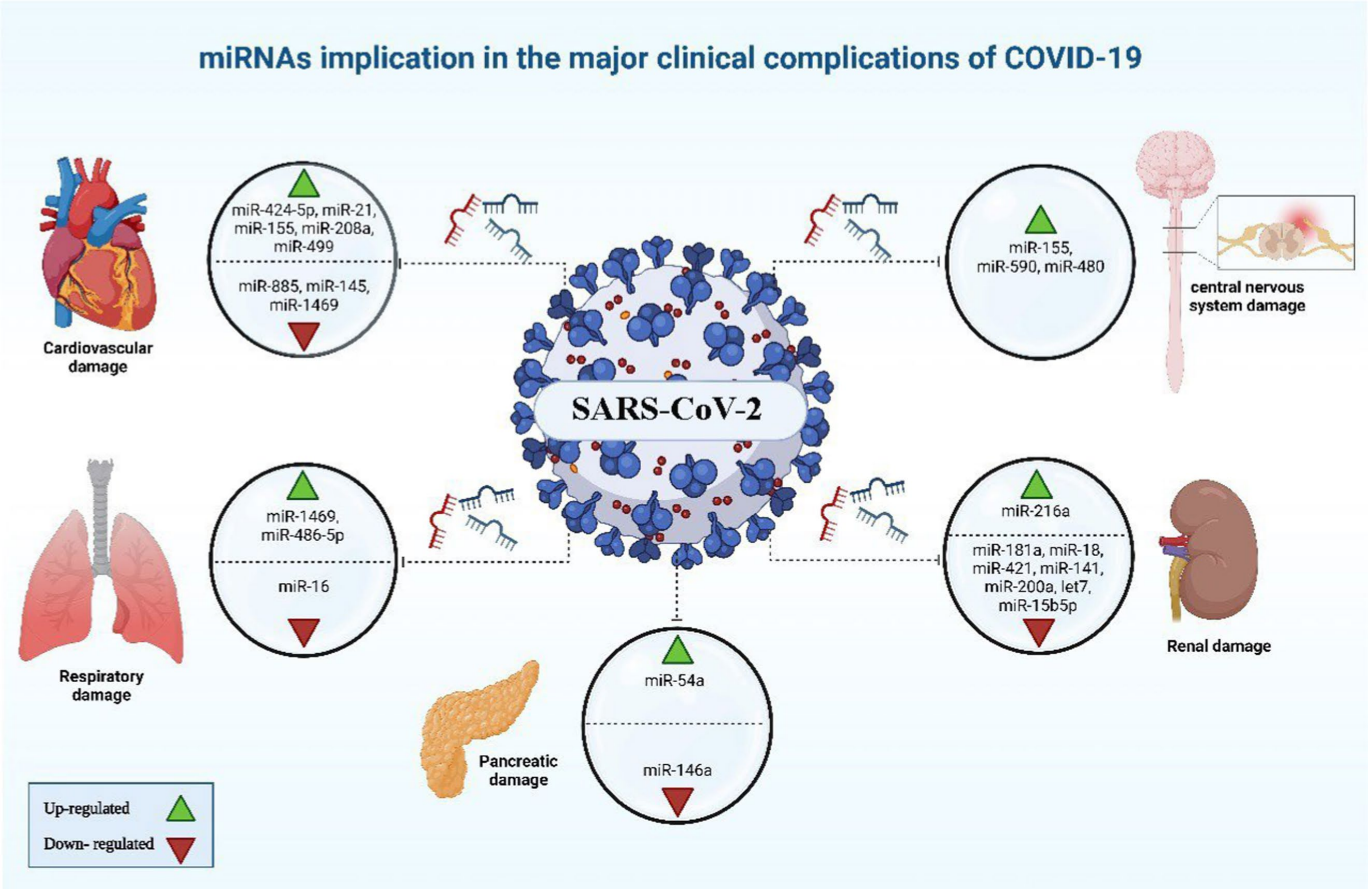
miRNAs implicated in the major clinical complications of COVID-19. The image was generated utilizing the BioRender software, accessible at the website https://www.biorender.com.

Oxidative stress arising when an excess of reactive oxygen species (ROS) overwhelm the body’s antioxidative mechanisms, resulting in cellular damage and potential tissue injury (87), acute lung injury and other pulmonary illnesses like chronic obstructive pulmonary disease are also caused by oxidative stress, beside its negative impact on cell communication. Research on human lung cell exosomes has shown that low oxygen levels cause a significant reduction in miR-3691-3p. This miRNA is vital for pathways associated with fibroblast growth factor 2 (FGF2), transforming growth factor-beta (TGF-β), and vascular cell adhesion molecule 1 (VCAM1) protein, that have importance in lung damage and repair (88). miR-146a and miR-486-5p level up IL-1β, IL-6, and TNF-α concentrations via the reduction of OTU domain-containing protein 7B (OTUD7B) during lung damage, beside stimulating NF-κB pathways, in which the latter leads to an elevated pulmonary tissue inflammation. Conversely, the overall inflammatory response is dampened, and the severity of acute lung injury is mitigated when PI3K, TNF-α, and IL-6 are downregulated by miR-16-5p (Figures 5, 6) (38).

###### 3.1.2.1.2 microRNA and its implications in cardiovascular problems and thrombosis

Certain patients infected with COVID-19 have been observed to develop cardiovascular complications (89). It was noted that COVID-19 cases with cardiovascular comorbidity have had elevated expression concentrations of miR-155, miR-499, miR-21, and miR-208a, acting like predictive factors for inflammation-induced myocardial damage (90). Furthermore, miR-208a demonstrated associations with arrhythmia, cardiac hypertrophy, and dilated cardiomyopathy. The mechanism underlying miR-208a-induced cardiac hypertrophy involves two pathways. Firstly, it induces the overexpression of medI3, leading to an increase in beta-myosin heavy chain (β-MHC) expression, a known hallmark of cardiac hypertrophy. Secondly, it enhances the expression of miR-499, which also contributes to the increase in β-MHC (Figures 5, 6) (91).

Alterations were observed in blood clotting indicators during the initial phase of COVID-19 pandemic, after measuring D-dimer, fibrinogen, and activated partial thromboplastin time (aPTT) of hospitalized patients by healthcare workers (92). Endothelial cells are pivotal in initiation and regulation of the thrombotic process, as they produce and release Von Willebrand factor and tissue factor. Impairment to the endothelium’s regular functionality as SARS-CoV-2 infects the body was hypothesized by researches, leading to system’s dis-balance. As a consequence, the synthesis and release of miR-885 and miR-145 by the endothelial cells is reduced, potentially leading to unregulated blood clots formation. Conclusions showed that there was a connection between the increased probability of thrombosis and mortality of COVID-19 cases with the low levels of exosomal miR-145, and miR-885 (93). Furthermore, the insufficiency of miR-146a and the elevation of miR-424-5p have also been linked to thrombosis in COVID-19 and could be considered as prognostic indicators for thrombotic conditions in COVID-19 (38).

###### 3.1.2.1.3 microRNA’s influence on COVID-19 neurological outcomes

The COVID-19 infection is notorious as a multi-organs illness, notably the central nervous system, persisting over a prolonged period, this situation is especially troubling for the elderly, who are more vulnerable to intense illness and are at higher risk of neurological conditions, regardless of the viral infection (67). miRNAs are prevalent throughout the central nervous system and exhibit unique patterns of expression based on location and time. Therefore, alternations to their typical expression levels may contribute to the development of neurological conditions (75). In neurological complications involving Alzheimer’s disease, down syndrome-related dementia, and multiple sclerosis, miR-155’s high concentrations contribute to inflammation. This type of miRNA mentioned earlier is found in association with high COVID-19 severity when found in elevated levels. It disrupts the blood-brain barrier, activates T cells, and fosters beta-amyloid plaque accumulation (67). Conversely, research indicated a decrease in many blood miRNAs during COVID-19 and different neurological issues. miR-21 as a common factor which plays its role in inflammatory reactions within the nervous system, regulation of normal cell death, in addition to its participation in glutamate-induced toxicity, activation of astrocytes, disruption in synapse function, increased activity of microglia, and restoration of myelin sheaths (94). Thus, miR-21 is critical in the progression and onset of diseases affecting the central nervous system. Hence, low levels of miR-21 lead to an amplification in the inflammatory response in COVID-19 pathogenesis due to its role as regulator of inflammation-related genes, this causes worsening of the disease’s state as well as influences neuro-inflammatory mechanisms (67). miR-148a specifically targets ubiquitin specific protein 33 (USP33), while miR-590 targets interferon regulatory factor 9 (IRF9). This targeting activates NF-κB, resulting in the overexpression of TNF-α and IFN-α, leading to severe neuro-inflammation (38). There was an observed elevation in miR-590 and miR-148a levels in cases with COVID-19 associated with neuro-inflammation (Figures 5, 6) (95).

###### 3.1.2.1.4 The impact of microRNA on renal diseases in COVID-19 context

Nephrons are found to express ACE2 receptors, thus, as SARS-CoV-2 cause renal impairment, it could be attributed to sepsis stemmed from cytokine storm in ill persons with severe stages of COVID-19, bringing about dehydration and could also reduce glomerular filtration rate (GFR). A correlation has been identified between SARS-CoV-2 infection, podocyte damage, and proteinuria (96). An increase in miR-216a, as well as missing let-7, miR-141, and miR-200a, serve as indication factors of kidney fibrosis during COVID-19, due to their potential in TGF-β expression activation (97). miR-181a, miR-18, and miR-421 have been linked to renal injury and the regulation of ACE2 expression (98). Diabetic nephropathy patients have a deficiency in miR-15b-5p, which is important for inhibiting apoptosis in renal cells which is provoked by the elevated levels of glucose via control of PTEN-PI3K/Akt pathway (Figures 5, 6) (99).

###### 3.1.2.1.5 The involvement of microRNA in metabolic syndromes linked to COVID-19

Individuals with conditions such as obesity, and diabetes commonly display diminished levels of a crucial regulator of inflammatory process which is miR-146a. miR-146a expression is induced in response to inflammation instigated by NF-κB, inhibiting TNF-α and IL-1 receptor activity to dampen the inflammatory response. Deficiency in miR-146 linked to diabetes leads to elevated inflammation from improper IRAK1/TRAF6 regulation (Figures 5, 6). SARS-CoV-2 can worsen COVID-19 severity by impairing miR-146a, leading to poor antiviral defenses, excess cytokine release, and uncontrolled inflammation-related tissue damage (66). On the other hand, the study by Khatami et al. (100) revealed an upregulation of miR-34a in COVID-19 patients with diabetes. Additionally, there was an association observed between the level of miR-34a and HbA1c (101).

##### 3.1.2.2 microRNAs in long COVID

Through systematic analysis of research data, it has been concluded that the predominant symptoms manifested by COVID-19 patients are as follows: fever, cough, fatigue, dyspnea, and sputum production (102). Headache, migraines, joint discomfort, muscle pain, and neuropathic pain sensation (4). When the pandemic began, prolonged COVID-19 symptoms were often mistaken for post-traumatic stress resulting from the disease’s novelty, lack of treatment, and associated social isolation, delaying the identification and naming of long COVID (103). Post-acute sequelae of SARS-CoV-2 or Long-haul COVID-19 as other terminologies for long COVID (104), a condition in which individuals face ongoing health complications persisting well after recovery from initial COVID-19 infection, far exceeding the typical recovery timeframe (105). Current research has identified long COVID as a separate condition with diverse symptoms, including respiratory problems like breathlessness and chest pain, neurological challenges like headaches and fatigue, and other issues such as hair loss, taste loss, and hearing impairment (4). Research explains that reduced concentrations of the tight junction protein claudin-1 (CLDN1), which are critical for preserving the integrity of the blood-brain and blood-nerve barriers, have been correlated with the manifestation of neuropathic pain. Animal studies and preliminary human data indicate a correlation between decreased claudin-1 and neuropathic discomfort. In case of long COVID, the elevation of miR-155-5p, a known miRNA for its action in claudin-1 suppression, could lead eventually to persistent neuropathic pain by expression reduction of claudin-1 levels and consequently increasing barrier permeability (4, 106).

#### 3.1.3 Viral microRNAs as exogenous participants

Mirroring the structural and functional features of human miRNAs, SARS-CoV-2 genome has been found to encode virus’s own miRNAs which exhibit capability to change the host’s immune mechanism and manage inflammatory responses as the infection progresses (47). Host miRNAs that manage crucial processes such as kidney and heart development, metabolism, neuronal functions, insulin resistance are compromised by viral infections. These miRNAs’ reduction in cases with COVID-19 disrupts such essential bodily processes, with the impact strengthened in individuals with underlying conditions (3). Research shows that miRNAs related to SARS-CoV-2 can hinder protein synthesis by attaching to human mRNA, affecting key proteins such as hemoglobin subunits (beta and gamma-globin2), type 1 interferons, and olfaction proteins. This may lead to the oxygen transport problems, immune changes, and olfactory dysfunction observed in COVID-19 patients, due to viral miRNA-like effects (107). The insulin signaling pathway is hypothesized to be susceptible to modulation by virally encoded miRNAs, leading to potential vulnerability exacerbation to SARS-CoV-2 infection of persons with comorbidities, especially diabetes. This suggests that viral miRNAs could play a role in epigenetic control and may interact with the host organism’s native miRNAs (3).

## 4 The role of microRNAs: implications for diagnosis

The early identification, immediate patients’ isolation, quick response to worsening health, and proper oxygen supplements along with good nutrition can notably reduce the risks of COVID-19 mortality. A standard test used to detect the virus is the nasopharyngeal swab polymerase chain reaction (PCR), posing risks to those dealing with specimens due to potential viral exposure. Thus, blood analysis for the detection of circulating miRNAs offers a safer diagnostic alternative (64). Certain circulatory miRNAs, like those found circulating in urine, tears, serum, and plasma have shown resistance against enzymatic degradation, which exhibit unique patterns associated with specific diseases. They are offered as potential non-invasive biomarkers for conditions like COVID-19 and can be detected by liquid biopsy (38, 64). Studies suggest that miRNAs can be affected by this viral infection, offering promise for their use in many aspects as shown in Table 2, such as the detection of the disease (108). In a study, the peripheral blood of cases with COVID-19 beside healthy individuals underwent high-throughput sequencing. Results demonstrated upregulation of 35 miRNAs while the rest 38 were showing downregulation. miR-16-2-3p stood out with an upregulation showing a 1.6 times elevation in comparison to healthy persons, whereas miR-627-5p showed most reduction (109). In the cases of COVID-19, research depicted alterations in nine miRNA types including upregulated hsa-miR-3614-5p and hsa-miR-3614-3p, while the downregulated ones involved hsa-miR-17-5p, hsa-miR-93-3p, hsa-miR-28-5p, hsa-miR-106a-5p, hsa-miR-17-3p, hsa-miR-100-5p, and hsa-miR-181d-5p, these changes in expression levels are proposed as reliable biomarkers to separate COVID-19 cases from healthy members (110). In comparison to healthy individuals, research findings informed a reduction in miR-126 levels in COVID-19 patients (90).

**Table 2:** Diagnostic microRNAs.

Circulatory miRNAs	Expression	Model	Clinical sample	Study design	Role	Source
miR-16-2-3p	↑	Human	Peripheral blood	COVID-19 patients: (*n* = 10) Control group (*n* = 4)	Associates with neurological complications and thrombosis	(109)
miR-627-5p	↓	Human	Peripheral blood		During infection, it regulates the immune response and viral replication	
hsa-miR-17-5p	↓	Human	Peripheral blood		During infection, it regulates the immune response and viral replication	
miR-126	↓	Human	Serum	Discovery study COVID-19 patients (*n* = 18) Control group (*n* = 15) Validation study COVID-19 patients (*n* = 20) Influenza-ARDS group (*n* = 13) Control group (*n* = 32)	Protects the endothelial layer from damage	(90)
hsa-miR-3614-3p	↑	Human	—	PRJNA736437 dataset COVID-19 patients (*n* = 36) Control group (*n* = 11) PRJNA737991 dataset COVID-19 patients (*n* = 188) Control group (*n* = 68)	COVID-19 diagnosis	(155)
hsa-miR-3614-5p	↑	Human				
hsa-miR-106a-5p	↓	Human				
hsa-miR-17-3p	↓	Human				
hsa-miR-17-5p	↓	Human				
hsa-miR-93-3p	↓	Human				
hsa-miR-181d-5p	↓	Human				
hsa-miR-28-5p	↓	Human				
hsa-miR-100-5p	↓	Human				
hsa-miR-17-3p	↑	Human	Blood (serum)	COVID-19 patients Grade 1 (*n* = 21) Grade 2 (*n* = 20) Grade 3 (*n* = 20) Grade 4 (*n* = 21) Grade 5 (*n* = 21) Control group (*n* = 20)	The maturation of miRNA is impaired due to down-regulation of ribonuclease III (Dicer 1) level	(140)
miR-26a-5p miR-29b-3p miR-34a-5p	↓	Human	Lung biopsies	COVID-19 patients (*n* = 9) Control group (*n* = 10)	Deficiencies in them are linked to severe lung injury, as they regulate inflammatory and endothelial signaling	(156)

COVID-19, coronavirus disease-2019; hsa, homo sapiens, miR, microRNA; SARS-CoV-2, severe acute respiratory syndrome-coronavirus-2.

miRNAs play a crucial role in the diagnosis of COVID-19. These small non-coding RNA molecules are involved in the regulation of gene expression and have been found to be dysregulated in various diseases, including viral infections. In the case of COVID-19, studies have shown that specific miRNAs are differentially expressed in infected individuals compared to healthy controls. These miRNAs can be detected and quantified using techniques such as reverse transcription polymerase chain reaction (RT-PCR) or next-generation sequencing (NGS). By analyzing the expression levels of specific miRNAs, healthcare professionals can potentially identify individuals who are infected with COVID-19, even in the absence of symptoms. Furthermore, miRNA-based diagnostic tests have the potential to provide rapid and accurate results, making them valuable tools in the fight against the pandemic.

## 5 The role of microRNAs: implications for prognosis

Diversified biomarkers were tested in order to ascertain their prognostic value in prediction of COVID-19 severity, among those, laboratory ones included ILs, D-dimer, and CRP, while genetic biomarkers involved TMPRSS2, SNPs in ACE2, TNF-α, and IFN-γ (111–114). The utilization of molecular techniques relying on miRNAs has the potential to enhance risk evaluation and offer a direct strategy for informing clinical judgments regarding patient care, monitoring, and treatment.

In the light of the pandemic, it is of utmost importance to define biomarkers capable of forecasting the severity of the illness, thereby mitigating SARS-CoV-2-induced fatalities. Taking into account the fact that circulating miRNAs can act as non-invasive markers for viral diseases. Establishing a predictor of disease severity is of utmost importance, allowing for proper stratification of patients based on disease severity and progression. Therefore, employing miRNAs as prognostic markers for early mortality in severe COVID-19 patients may greatly influence therapeutic strategies and patient care. This enables early stratification and personalized intervention (Table 3).

**Table 3:** Prognostic role of microRNAs.

Circulatory miRNAs	Expression	Model	Clinical sample	Study design	Role	Source
miR-323a-3p	↓	Human	Plasma	Study (1) Critical COVID-19 (*n* = 36) Moderate COVID-19 (*n* = 43) Study (2) ICU survivors (*n* = 20) ICU non-survivors (*n* = 16)	Predictors of ICU mortality, distinguishing between survived and non-survived cases	(119)
miR-192-5p	↓	Human	Plasma		Predictors of ICU mortality, distinguishing between survived and non-survived cases	
miR-451a	↓	Human	Plasma		Predictors of ICU mortality	
miR-16-5p	↓	Human	Plasma		Biomarkers for identifying mortality risk in critical COVID-19 patients	
miR-155	↑	Human	Serum	Discovery study COVID-19 patients (*n* = 18) Control group (*n* = 15) Validation study COVID-19 patients (*n* = 20) Influenza-ARDS group (*n* = 13) Control group (*n* = 32)	Differentiation between COVID-19 cases and influenza-ARDS, prediction of inflammation and chronic myocardial damage	(90)
miR-155	↓	Human	Serum	Severe illness (*n* = 15) Mild illness (*n* = 22) Control group (*n* = 215)	Prediction of mortality in COVID-19 infection	(75)
miR-21-5p miR-146a-5p	↓	Human	Blood	Severe illness (*n* = 6) Mild illness (*n* = 6) Control group (*n* = 4)	Biomarker for the infection’s severity, regulation of host’s immune response	(123)
miR-155	↑	Human	Plasma	COVID-19 patients: (*n* = 150) Control group (*n* = 50)	Development of COVID-19 infection, and biomarker for the infection’s severity	(157)
hsa-miR-192-5p	↑	Human	Plasma	GAIT-2 population *n* = 935	Biomarking potential for vein thrombosis	(120)
miR-2392	↑	Human	Serum, urine	COVID-19 patients: (*n* = 10)	Biomarking COVID-19, negative influence on mitochondrial gene expression, increase inflammation, increase oxidative stress	(126)
miR-146a-3p miR-29a-3p miR-155-5p	↑	Human	PBMCs	COVID-19 patients: (*n* = 18) Control group (*n* = 15)	Biomarking COVID-19 infection, miR-29a-3p, and miR-146a-3p may differentiate acute from post-acute stages of COVID-19 infection	(9)
miR-483-5p miR-320b	↑	Human	Serum	Discovery phase COVID-19 patients *n* = 12 (*n* = 6 alive, *n* = 6 deceased) Validation phase COVID-19 patients *n* = 116 (*n* = 75 alive, *n* = 41 deceased)	Predictors for determining survival probability in COVID-19 cases, different expression between survivors and non-survivors	(127)
miR-98-5p	↓	Human cells	Endothelial cells	HMVEC-L and HUVEC	Regulates the transcription of TMPRSS2	(115, 116)

ARDS, acute respiratory distress syndrome; COVID-19, coronavirus disease-2019; ICU, intensive care unit; GAIT-2, genetic analysis of idiopathic thrombophilia 2; HMVEC-L, human lung microvascular endothelial cells; HUVEC: human umbilical vein endothelial cells; miR, microRNA; PBMCs, peripheral blood mononuclear cells; TMPRSS2, transmembrane serine protease 2.

Intensity of COVID-19 infection is negatively correlated to miR-98-5p (115). miR-98-5p exhibits antiviral properties by binding to the viral genome, thereby inhibiting its replication (116). Moreover, intensely affected cases of SARS-CoV-2 infection depicted reduced patterns of miR-451a, and miR-323-3p (117). The probability of COVID-19 related CS occurrence is positively associated with the downregulation of miR-451a hypothesis suggested the interaction between miR-323-3p and the SARS-CoV-2 viral genome, as it was previously proposed to have binding ability to influenza gene exhibiting antiviral effects (118).

Based on the study performed by de Gonzalo-Calvo et al. (119), two miRNAs namely miR-192-5p and miR-323-3p were proposed to serve as prognostic biomarkers for the prediction of mortality caused by COVID-19 infection in intensive care unit (ICU), distinguishing non-survivors from those who survived. ROC analysis was taken as a support for this conclusion. Moreover, the study observed a negative relationship between period of ICU stay and expression levels of miR-323-3p and miR-192-5p. A significant connection has been established between a diminished level of miR-192-5p and the development of deep vein thrombosis (120).

The research entailed cooperation with 19 ICUs situated in diverse regions of Spain that are associated with the CIBERESUCICOVID initiative. A total of 503 plasma samples were procured from the patient population. After quantifying circulating miRNAs in plasma, the researchers established a signature composed of four miRNAs. The implementation of this signature facilitated the classification of patients according to their risk of mortality within 48 h of their admission to the ICU. The predictors of ICU mortality among COVID-19 have been confirmed to be miR-192-5p, miR-323-3p, miR-16-5p, and miR-451a, as determined through Kaplan–Meier survival analysis and LASSO regression methods (121). A study indicated that miRNAs found in macrophages had decreased levels subsequent to the exposure to SARS-CoV-2’s S proteins (122).

In a study, two potential biomarkers for determining COVID-19 intensity have been recognized, namely miR-21-5p and miR-146a-5p (123). Further research on severe cases revealed variations in cardiovascular and inflammatory miRNAs levels. Following the infection with influenza A virus, the exacerbated acute respiratory distress syndrome (ARDS) related lung damage was found to be due to an increased concentrations of miR-155-5p. It is suggested that miR-155 could be used as a potential therapeutic target given that its removal can mitigate pulmonary damage, particularly in COVID-19 illness (124). An additional beneficial attribute of miRNAs is their ability to serve as predictive biomarkers for tracking patient’s disease progression and to categorize patients, enabling health practitioners to offer more accurate and customized therapies (64). As for differentiating between severe from mild COVID-19 cases, research demonstrated that key clinical markers like C-reactive protein (CRP) and D-dimer have been outperformed by SARS-CoV-2-encoded miRNA called miR-nsp3-3p, additionally, its ability in distinguishing cases at higher risk even before the appearance of severe signs, rendered it as a prognostic indicator. This biomarker can be easily incorporated in COVID-19 diagnostic strategies due to the simplified measuring techniques (125). A study attempted to show how miR-2392 is involved in making it easier to become infected with SARS-CoV-2, it was concluded that this miRNA type negatively impacts the mitochondrial gene expression, beside its influence on inflammatory responses and glycolytic activity, relating to the poor outcomes and COVID-19’s symptoms, miR-2392 is found circulating in fluids like blood and urine of infected persons, it is shown to have an antiviral effect when reduced, as it influences severity of COVID-19 and how the disease progress (126). Distinguishing acute stage from the subsequent post-acute one as the COVID-19 progress was found to be possible by the utilization of hsa-miR-29a-3p and hsa-miR-146a-3p (9). Heightened concentrations of miR-320b and miR-483-5p are positively linked to higher risk of mortality, these two miRNAs can serve in determination of survivability in COVID-19 cases, acting like crucial predictors. The research investigating their role, indicated that patients with the highest concentrations of these miRNAs were three times more likely to die during hospitalization (127). These findings offer potential for improving patient care and creating specialized treatments.

The role of miRNAs in prognosis is of utmost importance as they have been implicated in various diseases, including cancer, cardiovascular diseases, and viral infection. By modulating gene expression, miRNAs can have profound effects on cellular processes such as cell proliferation, differentiation, and apoptosis. Furthermore, dysregulation of miRNA expression has been associated with disease progression and poor prognosis in many cases. Therefore, understanding the prognostic implications of miRNAs can provide valuable insights into disease progression, patient outcomes, and potentially guide treatment decisions for improved personalized medicine approaches.

## 6 Implications of microRNAs for therapeutics

In addition to the utilization of vaccines and repurposed drugs for COVID-19 prevention and treatment, scientists are also exploring novel therapeutics like miRNA (128). The growth of miRNA-derived drugs is rapidly occurring and has the potential to revolutionize standard treatments for various diseases, including COVID-19. They offer distinct benefits when compared to traditional small molecule and biologic medicines. These include cost-effectiveness, ease of manufacture, and the ability to target previously unresponsive sites. Through the implementation of chemical modifications and RNA nanocarriers, it is possible to alleviate or minimize the typical challenges associated with stability, delivery, and undesired effects.

Variations in miRNAs profiles are indicative of COVID-19 severity, with notable implications in patients with comorbidities, suggesting that targeting such altered non-coding RNAs can attenuate the complications (98). Manipulation of miRNAs, like those mentioned in Table 4, might attenuate COVID-19’s adverse effects by the inhibition of chemokines and cytokines release, potentially leading to more favorable outcomes. miRNAs could be an effective treatment strategy among the other nucleic acid approaches for addressing COVID-19 (129). Hindering viral attachment offers an effective early strategy to combat COVID-19, by targeting miRNAs regulating proteins such as ACE2 and TMPRSS2 important for viral entry into host cells (98). A therapeutic strategy that harnesses antiviral beside anti-inflammatory miRNAs could be more efficacious than a single-agent therapies, targeting miRNAs, essential proteins, or the viral genome offering a hopeful strategy in the development of disease treatments (98).

**Table 4:** microRNAs as targets for COVID-19 therapeutic development.

microRNAs	Biological effect	Source
miR-200b/c	Antagonist, regulates expression of ACE2 protein, reduce ACE2-mediated infection	(158)
miR-200c-3p	Antagonist, regulates expression of ACE2 protein	(159)
miR-24-3p miR-145-5p miR-7-5p miR-223-3p	Antagonists, have ability to hinder the expression of S proteins of SARS-CoV-2 and inhibiting viral replication	(68)
miR-155	Antagonist (cobomarsen), restores normal expression of dysregulated proteins of the host	(130, 160)
miR-29	Agonist, restores normal expression of dysregulated proteins of the host	(130)
hsa-miR-146a-5p	Agonist, predicts response to TCZ, Low serum levels were noted in patients with COVID-19 un-responsive to TCZ	(133)
miR-421	Antagonist, regulates ACE2 expression post-transcriptionally	(161)
miR-16	Agonist, induces apoptosis through the downregulation of the BCL2	(162)
miR-24	Agonist, regulates furin	(137)

ACE2, angiotensin-converting enzyme 2; miR, microRNA; S, spike; SARS-CoV-2, severe acute respiratory syndrome-coronavirus-2; TCZ, tocilizumab.

Therapeutic strategies have been emerging lately, in which miRNAs (antagonists or inhibitors) are used to target host’s miRNAs, reducing the negative impact of such native miRNAs. miR-155 inhibitor called cobomarsen and miR-29 mimic with the name remlarsen have the potentiality to restore normal expression of dysregulated proteins of the host. Thus, anti-miRs and miR mimics may serve as therapeutic options for managing COVID-19 disease (130). Extracellular vesicles (EVs) are investigated as a promising therapeutic approach due to their cell-free nature, being immunologically safe, and lack exogenous substances (131). Cells release membrane-bound structures called EVs, which encapsulate genetic components such as miRNAs, DNA, and long non-coding RNA (lncRNA), as well as other biologically active molecules derived from the parental cells (132). Two analytical methods, quantitative real-time PCR along with high-throughput sequencing indicated four miRNA types miR-24-3p, miR-223-3p, miR-145-5p, and miR-7-5p that demonstrated their ability in impeding SARS-CoV-2’s S proteins and the inhibition of viral replication either in the free or exosomal form as observed in serum of young persons, these miRNAs showed a significant reduced pattern in the elderly and those with diabetes (68). While further investigating the potentiality of miRNAs as biomarkers, it was found that specific miRNAs found within the circulatory system can serve as predictive indicators for a patient’s response to medicines. COVID-19 patients who did not respond to tocilizumab (TCZ) exhibited diminished serum levels of hsa-miR-146a-5p (133).

It is of interest to mention that miR-421 has been implicated in the regulation of ACE2, highlighting its relevance in therapeutic investigations for SARS-CoV-2 (134). miR-16 possesses additional therapeutic potential due to its role in inducing apoptosis through the downregulation of the BCL2 (135). Apoptosis plays an important role in the initial antiviral defense by prompting the clearance of virally infected cells. For instance, in the context of influenza infection in mouse cells, the induced apoptotic response facilitates the removal of infected cells by phagocytic action of phagocytes as macrophages and neutrophils. However, in the case of SARS-CoV-2, the virus can evade this innate immune pathway by downregulating miR-16 leading to the inhibition of the process of apoptosis in host cells. Moreover, it was reported that inhibition of miR-16 leads to elevated production of mitochondrial ROS as well as upregulation of TLR4 expression (136). Thus, the possibility of upregulating miR-16, potentially through the use of a miRNA mimic, emerges as a viable strategy to counteract the deleterious manifestations of COVID-19 (137).

Severe COVID-19 cases exhibit an upregulation of miR-200b/c. The reduction of ACE2 is the mechanism by which this miRNA exerts its detrimental effects. Therapeutic targeting can be achieved by blocking miR-200b/c. The decline in ACE2 levels is deleterious for the control of pulmonary fibrosis because ACE2 is key in metabolizing Ang II, a peptide hormone which is recognized for instigating lung fibrosis by stimulating TGF-β, and without sufficient ACE2, this harmful process is exacerbated. Elevated miR-200c-3p is associated with diminished ACE2 expression in H5N1 viral infection, potentially leading to lung injury. A similar reduction in ACE2 levels has been noted in infections by SARS-CoV-2 and the earlier SARS-CoV-1, resembling the H5N1 viral impact. Additional practical studies, both *in vivo* and *in vitro* are necessary to define the expression patterns of miR-200b/c throughout infections of SARS-CoV-2 and SARS-CoV-1. Research has elucidated the pivotal role of ACE2 in regulating heart function, indicating a potential correlation between the accumulation of Ang II and the initiation of cardiovascular diseases. Further studies elucidating the regulatory effect of miR-200b/c on ACE2 could potentially illuminate the cardiac complications observed in COVID-19, including acute coronary syndrome, heart failure, arrhythmias, and heart inflammation.

The serine protease furin plays a crucial role in facilitating the entry of enveloped respiratory viruses like MERS-CoV, H5N1, and SARS-CoV-2, into host cells by mediating the proteolysis of surface glycoproteins necessary for virus membrane attachment and fusion. It is implicated that miR-24 can regulate this process. Furin exhibits widespread expression across various tissues, including those within the respiratory system, making it a key factor for these viruses to exploit for successful infection and pathogenesis. Enzymatic adjustments are key to prompting membrane adherence and integration, impacting the virus’s cell tropism and pathogenic potential. The presence of a furin-like cleavage site (FCS) on the S protein of SARS-CoV-2, which is not identified in SARS-CoV-1, is crucial for the virus’s ability to bind and fuse with host cell membranes. This site might be integral to the enhanced membrane fusion capabilities that are a hallmark of SARS-CoV-2’s viral pathogenicity and infectivity. During SARS-CoV-2 infection, a decrease in miR-24 levels may lead to increased expression of furin, facilitating viral entry and replication. Additionally, the consequent activation of transforming growth factor beta 1 (TGF-β1) by furin could worsen the injury of the lung by prompting the progression of pulmonary fibrosis, which is prevalent in intense COVID-19 cases.

Mesenchymal stem cells (MSCs) giving rise to EVs express a protective service against SARS-2 coronavirus, mitigating cellular damage by lessening inflammatory response through viral spreading hindrance. Proposed as a possible treatment for COVID-19, mesenchymal stem cells are currently under examination, owing to their capacity improving the lung damage repair, as observed in pneumonia cases, in addition to their ability to stimulate macrophages to devour pathogens, successfully limiting viral spread (138). Efficient and safe delivery of miRNAs to a specific desired location within the body is slowed down by challenges such as direct targeting, stability, possibility of side effects. This highlights the importance of employing suitable delivery mechanisms to facilitate the reliable transfer of miRNAs for therapeutic purposes (98). The usage of MSCs or their derivatives is considered as a possible therapeutic approach and suitable carriers for delivering miRNAs owing to their tendency to be drawn to damaged or inflamed areas, a phenomenon known as tropism (98, 139).

The implications of miRNAs for therapeutics are highly significant. Due to their ability to target multiple genes simultaneously, miRNAs have emerged as potential therapeutic targets for various diseases, include COVID-19. By modulating the expression of specific miRNAs, it is possible to manipulate the expression of target genes and potentially correct aberrant gene expression patterns associated with disease. This opens up new avenues for the development of novel therapeutic strategies that can specifically target disease-associated pathways and potentially offer more effective and precise treatments. Furthermore, the stability and accessibility of miRNAs in body fluids, such as blood and urine, make them attractive as potential biomarkers for monitoring treatment response. Therefore, understanding the implications of miRNAs in therapeutics holds great promise for the development of personalized medicine and improved patient outcomes.

### 6.1 miRNA can be used as a marker to monitor treatment outcomes and drug response

miRNAs have the advantage of being potential multi-marker models for guiding treatment and assessing treatment response. The process of running multiple protein markers during research can be both expensive and time-consuming. However, using different sets of miRNA panels could provide a non-invasive method for tracking the effectiveness of drugs. There has been significant research conducted on the relationship between miRNAs and drug resistance. Tocilizumab has not been effective in treating COVID-19 cases with low levels of miR-146a (133). COVID-19 patients who were hospitalized and showed increased levels of miR-29a, miR-31, and miR-126 did not exhibit any response to treatment. On the other hand, the patients who showed a positive response to the treatment exhibited those miRNA levels within the normal range (140).

### 6.2 Enhancing therapeutic approaches: nutraceutical targeting of microRNAs modulating inflammation in the context of COVID-19

An alternative strategy to combat COVID-19 infection involves the employment of natural compounds in the treatment process. Inadequate nutritional status can lead to oxidative stress and inflammation, affecting the immune system’s regular functions. This review focuses on the use of polyphenol as a nutraceutical for therapeutic purposes. Polyphenol is an ideal example for several reasons, primarily because it is found abundantly in commonly consumed foods such as fruits, vegetables, and tea (141). Additionally, the mechanism of action is clearly identified against numerous viruses (142). Lastly, it’s important to note that this review does not specifically focus on the role of secondary metabolite modulation of miRNA in SARS-CoV-2 infection; polyphenol is simply used as an example. Owing to their capabilities, polyphenols can modify signals within cells and the expression of genes that are linked to oxidative stress. This capability indicates a potential impact on the severity of infections caused by SARS-CoV-2 (143). Research has demonstrated that polyphenols can combat viruses directly by damaging the pathogens or enhancing the adaptive immune response indirectly, according to both laboratory and living organism studies. A comparison between two human miRNA sets was done. Analysis demonstrated interaction of 17 miRNAs with the SARS-CoV-2 genome and are also modulated by polyphenols, including hsa-miR-148a-5p, hsa-miR-25-5p, hsa-miR-320c, and hsa-let-7a-3p (144). Quercetin, epigallocatechin-3-gallate (EGCG), resveratrol, and curcumin, are four polyphenols often utilized as nutritional therapeutic agents, showing their efficiency as prophylaxis of SARS-CoV-2 infection (64), having the following effects summarized in Table 5.

**Table 5:** A glance into the effect of polyphenols on SARS-CoV-2 infection by modulating microRNAs (64).

Types of polyphenols	Biological impact on SARS-CoV-2
Quercetin	Daily nutritional intake can hinder viral infection by altering the expression of certain miRNAs, including members of the let-7 family, hsa-miR-16, and the hsa-miR-200 group
EGCG	Inhibits the entry of SARS-CoV-2 into host cells and decrease the synthesis of S proteins by modulating specific miRNAs like members of the let-7 group, hsa-miR-15b, hsa-miR-125b, and hsa-miR-497-5p Treatment with EGCG significantly elevated the levels of hsa-miR-3613-3p, which is thought to obstruct the replication of the SARS-CoV-2 virus by binding to the viral 3′-UTR, interfering with its RNA sequences
Resveratrol	Halts the multiplication and spread of the virus by leveling up miRNAs from the hsa-miR-200 family, and hsa-miR-125b expression, leading to reduced ACE2 receptors’ level. Furthermore, hsa-miR-15b and hsa-miR-622 play a role as they bind to viral S proteins to hinder the infection
Curcumin	Induction of miRNAs from let-7 group, in which the latter inhibit TMPRSS2 expression, can hinder viral entry by preventing the virus from accessing host cells Curcumin intake leads to upregulation of hsa-miR-15b, members of the hsa-miR-200 group, and hsa-miR-125b, which in turn downregulate the gene expression of ACE2 receptors and the viral S proteins

ACE2, angiotensin-converting enzyme 2; EGCG, epigallocatechin-3-gallate; hsa, homo sapiens; miR, microRNA; S, spike; TMPRSS2, transmembrane serine protease 2.

### 6.3 The interaction between vaccines, variants of concern, and miRNA

Vaccines, variants of concerns (VOCs), and miRNA have an intricate relationship. Vaccines aim to enhance the immune system’s response against the SARS-CoV-2 virus, while VOCs suppress the immune system in order to ensure their own survival. mRNA vaccines like Mrna-1273 and BNT162b utilize the host machinery of miRNA to combat viruses. miRNA micro-arrays are employed to determine the effectiveness of COVID-19 vaccines (145). Adverse reactions to COVID-19 vaccines and antibody levels can be determined by examining extracellular vesicle (EV) miRNAs like miR-92a and miR-148a. These miRNAs are utilized as biomarkers to evaluate the effectiveness and safety of vaccines (146). The presence of miRNAs like has-miR-4784 and has-miR-3150b-3p decreases the binding of Omicron strains to their receptors and diminishes the virulence of the virus (147).

## 7 Recommendations and limitations for microRNA research in COVID-19

### 7.1 Recommendations

#### 7.1.1 Utilize miRNA for therapeutic purposes

Additional research is needed to test miRNAs in cell culture, animal models, and humans to inhibit SARS-CoV-2 binding, replication, or apoptosis in infected cells. Clinical trials should test miRNA mimics or anti-miRNA for treating viral infections. Testing miRNAs in combination with antiviral or immunomodulatory drugs could provide a synergistic approach to the treatment of COVID-19. By targeting both the viral replication and the dysregulated immune response, this combination therapy could potentially enhance the efficacy of treatment and reduce the severity of the disease. Further research and clinical trials are needed to evaluate the safety and effectiveness of miRNA-based combination therapies for COVID-19.

#### 7.1.2 Analysis of additional miRNA as prognostic and diagnostic markers

More miRNA should be examined for high area under curve (AUC), specificity, and sensitivity. Additionally, miRNA levels should be assessed at various stages of COVID-19 to predict disease severity.

The identification of reliable biomarkers for early prediction and diagnosis of long COVID-19 (post-acute sequelae of COVID-19) is crucial for effective management and intervention. In recent years, miRNAs have gained attention as potential biomarkers due to their stability and role in regulating gene expression. Therefore, assessing the use of miRNAs as potential predictors of long COVID-19 could provide valuable insights into the pathophysiology of the disease and aid in the development of personalized treatment strategies.

### 7.2 Limitations

Using miRNA is limited by the dynamic nature of miRNA expression. The levels of miRNA can fluctuate over time, making it challenging to establish a reliable baseline for diagnostic or prognostic purposes. Additionally, the expression patterns of miRNA can be affected by various factors such as age, sex, comorbidities, and even medication use. Moreover, the genetic background of individuals plays a significant role in miRNA expression, as certain genetic variations can impact the processing and stability of miRNAs. All these factors contribute to the variability in miRNA expression, making it difficult to establish a universal miRNA signature for COVID-19 diagnosis or prognosis. However, despite these limitations, miRNA profiling still holds promise as a potential biomarker tool in COVID-19 research, especially when integrated with other clinical and molecular data. Ongoing studies are focusing on developing standardized protocols and analytical methods to overcome the challenges posed by miRNA variability and harness their potential as reliable diagnostic and prognostic markers in COVID-19.

The complexity of miRNA-target interactions poses a limitation to miRNA utilization. miRNAs often exhibit pleiotropic effects, meaning that a single miRNA can target multiple genes simultaneously. While this ability allows miRNAs to regulate various biological processes, it also increases the risk of potential side effects. When a miRNA targets multiple genes, the regulation of one gene may inadvertently affect the expression or function of other genes. This can lead to unintended consequences and disrupt normal cellular processes. Therefore, the pleiotropic effects of miRNAs must be carefully considered when studying their functions or developing therapeutic interventions involving miRNA-based therapies. Understanding the complex network of miRNA-gene interactions is crucial for predicting and minimizing potential side effects associated with miRNA-based treatments.

The method of delivery of miRNA to the target site is another significant limitation that researchers face in the field of miRNA therapeutics. Currently, there are various approaches for delivering miRNA molecules to specific tissues or cells, including viral vectors, liposomes, nanoparticles, and exosomes. However, each of these delivery methods has its own challenges and drawbacks. For instance, viral vectors can induce immune responses and have limited cargo capacity. Liposomes and nanoparticles may suffer from poor stability, low efficiency, and potential toxicity. Exosomes, on the other hand, have limited loading capacity and lack precise control over delivery. Therefore, finding an efficient and safe delivery system for miRNA remains a critical hurdle in realizing the full therapeutic potential of miRNA-based therapies. Efficient and targeted delivery of miRNA mimics or inhibitors remains a significant hurdle. Current delivery methods may not ensure the stability and bioavailability of these molecules, which is essential for their therapeutic efficacy.

One limitation of the few ongoing clinical trials for investigating the safety and efficacy of miRNA as therapeutics is the small sample size. Due to the novelty and relatively recent discovery of miRNA as potential therapeutic targets, there are currently limited clinical trials available. This small sample size can limit the generalizability of the results and make it difficult to draw definitive conclusions about the safety and efficacy of miRNA therapeutics. Additionally, the lack of diversity in the study populations can further restrict the applicability of the findings to a broader range of patients. Furthermore, the limited number of clinical trials may also hinder the identification of potential adverse effects or long-term consequences associated with miRNA therapeutics. Therefore, while the ongoing trials provide valuable insights into the potential benefits of miRNA therapeutics, the limited number of studies is a notable limitation that needs to be addressed in future research. Additionally, the role of certain miRNA in relation to COVID-19 remains unknown. Ultimately, to effectively study miRNA, one must have access to advanced bioinformatics tools and a large dataset.

## 8 Conclusion

To recap, this comprehensive analysis clarifies the intricate role of miRNAs in the pathogenesis, diagnostic frameworks, prognostic assessments, and therapeutic interventions in relation to COVID-19, underscoring their significance as regulators in the interplay between host and virus, influencing immune responses, inflammatory processes, along with viral replication. Moreover, miRNAs act as potent regulatory molecules of gene expression, exerting control by attaching to compatible interaction sites. Many clinical targets for COVID-19 are miRNAs, proposing that the virus might manage its entry into cells and disrupt bodily functions by altering these regulatory miRNAs. Both host-and virus-derived miRNAs play a critical role in helping the virus to evade immune system’s components, triggering inflammatory responses, leading to overproduction of cytokines, and tissue damage. Shifts in miRNAs’ concentration may potentially mark variable stages of COVID-19 illness. Furthermore, it was discovered that miRNAs performed better in terms of diagnosis than frequently used assays. Despite showing the impact that the host-and viral-encoded miRNAs have on the development of COVID-19 in several investigations, but the actual practical usage of such findings regarding the treatment and prevention are still in the early developmental stages.
